# Editorial: Sudden infant death syndrome: Moving forward

**DOI:** 10.3389/fped.2022.972430

**Published:** 2022-07-20

**Authors:** Christèle Gras-Le Guen, Patricia Franco, Sabine Plancoulaine

**Affiliations:** ^1^Département de Pédiatrie, Centre Hospitalier Universitaire de Nantes, Nantes, France; ^2^INSERM U1028 Centre de Recherche en Neurosciences de Lyon, Bron, France; ^3^Hôpital Femme-Mère-Enfant, Hospices Civils de Lyon, Bron, France; ^4^Université Paris Cité, Inserm, INRAE, Centre de Recherche en Epidémiologie et StatistiquesS (CRESS), Paris, France

**Keywords:** sudden infant death syndrome (SIDS), sudden unexpected death in infancy (SUDI), epidemiology, pathophysiology, intervention

Sudden unexpected death of infants (SUDI) remains a tragedy for hundreds of parents, siblings and families in 2022. It is still one of the leading causes of death between 28 and 360 days of age in high-income countries (1).

Infant mortality is a well-known key indicator of population health. It is strongly related to socio-economic development, quality of preventive and curative care. However, the incidence of SUDI differs greatly among western countries, with great heterogeneity and no evident explanations (2). In the 1990s, great hope was born with the description of the prone sleeping position as a main risk factor of SUDI. However, although the back-to-sleep campaign initially had spectacular impact in limiting death, since 2000, the incidence has stagnated at a too-high level. Some complementary risk factors have been reported since this period, with changeable factors (e.g., sleeping environment, tobacco exposure) as well as non-changeable factors (e.g., sex, prematurity). These different risk factors were combined in the triple risk hypothesis in 1994 (3).

Thirty years after the prone sleeping risk-factor discovery, it is now urgent to investigate new risk factors and understand the current pathophysiology of SUDI to find complementary prevention measures and build large programs to limit avoidable deaths.

In this Research Topic, we are pleased to gather multi-approach research papers with new perspectives and promising international collaborations. The publications provide new insights into epidemiological data and more specifically age-specific risk factors (Kanits et al.); breastfeeding and sleeping practices in The Netherlands, with a warning on a new increase in unsafe habits in the last decade (Kanits et al.); and the mis-opportunity of preventive care in the days before sudden infant death syndrome (SIDS). Indeed, more than 25% of SUDI cases had a healthcare encounter within 7 days of their death, and notably, all unsafe sleep behaviors had increased in frequency before and during the SUDI event as compared to routine (Salada and Badke). This is also a warning for the increased risk of SIDS when there is a combination of maternal alcohol, tobacco, and recreation drug use and bed sharing (Hauck and Blackstone). We also published a literature review on the interventions for safer sleep practices showing that information-exchange personalized models are the most effective interventions (Ellis et al.). Concerning the pathophysiological mechanisms, anatomical characteristics such as ogival palate is frequently observed in SUDI, which suggests chronic obstruction as an additional risk factor (Ducloyer et al.) Also, at the molecular level, the serotoninergic system and maturation of histaminergic systems (Plancoulaine et al.) have been deciphered, with different subtypes of observed cardiorespiratory and arousal deficits (Haynes et al.). Another review reports that thermal stress can alter cardiovascular and respiratory functions and lead to life-threatening events (Bach and Libert). These functions could be explored with new tools to assess newborns' autonomous reactivity and identify children at high risk of SIDS (Patural et al.).

A multitude of questions still remain.

1) What pathophysiological mechanisms and pathways are involved?
2) Why are there such geographic variations in SUDI incidence?
3) What is the relative responsibility of genetics, infections, toxicological or drug exposure?
4) Why do so many infants still sleep in the prone position 30 years after back-to-sleep campaigns? What are the current breaks in guidelines' implementation and how can we improve their acceptability?
5) What place do biomarkers, medical software solutions, and health digital learning have in prediction and prevention?
6) What are the perspectives for consolidating and developing international registers and collaborative databases?
And many others …

SUDI today must be considered a priority Research Topic and involve physicians, scientists, teachers, childcare professionals and family associations altogether. SUDI should no longer be a fatality but rather become a preventable accident with evidence-based prevention measures shared by all child carers.

## Author contributions

CG-L, PF, and SP contributed to the redaction of this editorial and approved the submitted version.

## Conflict of interest

The authors declare that the research was conducted in the absence of any commercial or financial relationships that could be construed as a potential conflict of interest.

## Publisher's note

All claims expressed in this article are solely those of the authors and do not necessarily represent those of their affiliated organizations, or those of the publisher, the editors and the reviewers. Any product that may be evaluated in this article, or claim that may be made by its manufacturer, is not guaranteed or endorsed by the publisher.
